# Incidence and origin of histologically confirmed liver metastases: an explorative case-study of 23,154 patients

**DOI:** 10.18632/oncotarget.10552

**Published:** 2016-07-13

**Authors:** Jannemarie de Ridder, Johannes H. W. de Wilt, Femke Simmer, Lucy Overbeek, Valery Lemmens, Iris Nagtegaal

**Affiliations:** ^1^ Department of Surgical Oncology, Radboud University Medical Center, Nijmegen, The Netherlands; ^2^ Department of Pathology, Radboud University Medical Center, Nijmegen, The Netherlands; ^3^ Foundation PALGA, Nationwide Network and Registry of Histo- and Cytopathology in the Netherlands, Utrecht, The Netherlands; ^4^ Netherlands Cancer Registry, Comprehensive Cancer Organisation the Netherlands (IKNL), Eindhoven, The Netherlands; ^5^ Department of Public Health, ErasmusMedical Center, Rotterdam, The Netherlands

**Keywords:** liver metastases, histology, incidences, colorectal liver metastases, non-colorectla livermetastases

## Abstract

**Background:**

The liver is a common metastatic site for a large variety of primary tumors. For both patients with known and unknown primary tumors it is important to understand metastatic patterns to provide tailored therapies.

**Objective:**

To perform a nationwide exploration of the origins of histological confirmed liver metastases.

**Results:**

A total of 23,154 patients were identified. The majority of liver metastases were carcinomas (n=21,400; 92%) of which adenocarcinoma was the most frequent subtype (n=17,349; 75%). Most common primary tumors in patients with adenocarcinoma were from colorectal (n=8,004), pancreatic (n=1,755) or breast origin (n=1,415). In women of 50 years and younger, metastatic adenocarcinoma originated more frequently from breast cancer, while in women older than 70 years liver metastases originated more frequently from gastrointestinal tumors. Liver metastases in men older than 70 years originated often from squamous cell lung carcinoma. An unknown primary tumor was detected in 4,209 (18%) patients, although tumor type could be determined in 3,855 (92%) of them.

**Methods:**

Data were collected using the nationwide network and registry of histo- and cytopathology in the Netherlands (PALGA). All histological confirmed liver metastases between January 2001 and December 2010 were evaluated for tumor type, origin of the primary tumor and were correlated with patient characteristics (age, gender).

**Conclusion:**

The current study provides an overview of the origins of liver metastases in a series of 23,154 patients.

## INTRODUCTION

The liver is a common site for metastatic disease, however, little is known about the frequency in which various tumors present with liver metastases. Understanding these metastatic patterns is important for patients with a known primary tumor, as well as for patients with an unknown primary tumor. Knowledge of preferred metastatic sites in the first group of patients may direct staging and surveillance schemes, while in the group of patients with an unknown primary tumor the patterns can be used to predict the primary tumor site, which is important for treatment.

The high frequency of liver involvement in metastatic disease can be explained by the different hypotheses of metastatic spread. The double blood supply of the liver by the portal vein and the hepatic artery facilitates entrapment of circulating cancer cells, according to the “*mechanical or hemodynamic hypothesis*” [1], which explains the high incidence of liver metastases in patients with gastrointestinal carcinomas. However, some primary tumors selectively target the liver as a metastatic location, according to the *“seed-and-soil” hypothesis* [2]; examples are patients with uveal melanoma with a loss of chromosome 3 [3], and patients with breast cancer with the human growth factor receptor 2 (HER-2) positivity in combination with estrogen (ER) and progesterone receptor (PR) positivity [4].

Metastatic patterns in colorectal cancer have recently been evaluated in a large nationwide autopsy study, describing all autopsies between 1991-2010 [5]. Hugen et al. demonstrated development of liver metastases in 32%-73% of the colorectal cancer patients, with significant differences between various histological subtypes [5]. While it is known that the majority of the liver metastases are of colorectal origin, exact data about incidences of non-colorectal liver metastases are scarce.

Large scale autopsy studies could potentially provide information, but these studies are rare, and often based on much older cohorts [6, 7]. An example is the study of DiSibio *et al.* which describes a cohort of autopsies between 1914 and 1943 [6]. Since 1943 changes in both surgical treatment and adjuvant therapy are profound and likely to have influenced detection and development of liver metastases. To date, it remains unclear which primary tumors, other than colorectal cancer, metastasize to the liver and in which frequency they do so.

By analyzing all liver biopsies in an era of modern diagnostics and treatments, the incidences of liver metastases can be estimated for different primary tumors. This large scale, systematic, nationwide analysis of pathology reports generated between 2001 and 2010 showed new insights into the origins of liver metastases.

## RESULTS

### General patient characteristics

During the study period, 24,136 pathology reports (20,098 liver biopsies and 4,038 liver resections) were retrieved. Double counts were excluded for 982 patients who underwent both a liver biopsy and liver surgery (*n*=390), patients who underwent multiple liver resections (*n*=342) or patients who underwent more than one liver biopsy (*n*=250). A total of 23,154 patients were included in the study (47% female). Median age at the time of liver biopsy was 67 years (range 0-97 years), and 63 years (range 1-91years) at the time of liver resection. The patients who underwent a liver biopsy at the age of 0 (*n*=3), were diagnosed with neuroblastoma, while the one-year old patient underwent a liver resection for metastatic Wilms tumor.

The amount of liver biopsies did not significantly increase over time. In 2001, 1,934 biopsies were performed, compared to 2,232 in 2010. In contrast, there was a significant decrease in pre-operative biopsies, from 10.8% in 2001 to 8.8% in 2010 (*p*<0.001). An increase of liver resections was observed; from 224 in 2001 to 596 in 2010 (*p*<0.0001).

### Tumor types and organs of origin

Carcinoma was the most frequent tumor type, diagnosed in 21,400 patients (92.4%), followed by melanoma in 547 patients (2.4%), and sarcoma in 235 patients (1.0%). In 33 patients (0.1%) the tumor type was classified as ‘other’. The pathologist was unable to define the tumor type of the liver metastases in 938 patients (4.1%). (For detailed information see Supplementary Table S1)

### Carcinoma

Adenocarcinoma not otherwise specified (N.O.S.) was the most frequent subtype of carcinoma (*n*=17,349; 74.9%), followed by small cell carcinoma (*n*=1357, 5.9%), neuroendocrine carcinoma (*n*=1072; 4.6%), large cell carcinoma (*n*=877; 3.7%), and squamous cell carcinoma (*n*=335; 1.4%). (See Supplementary Table S1).

**Figure 1:**
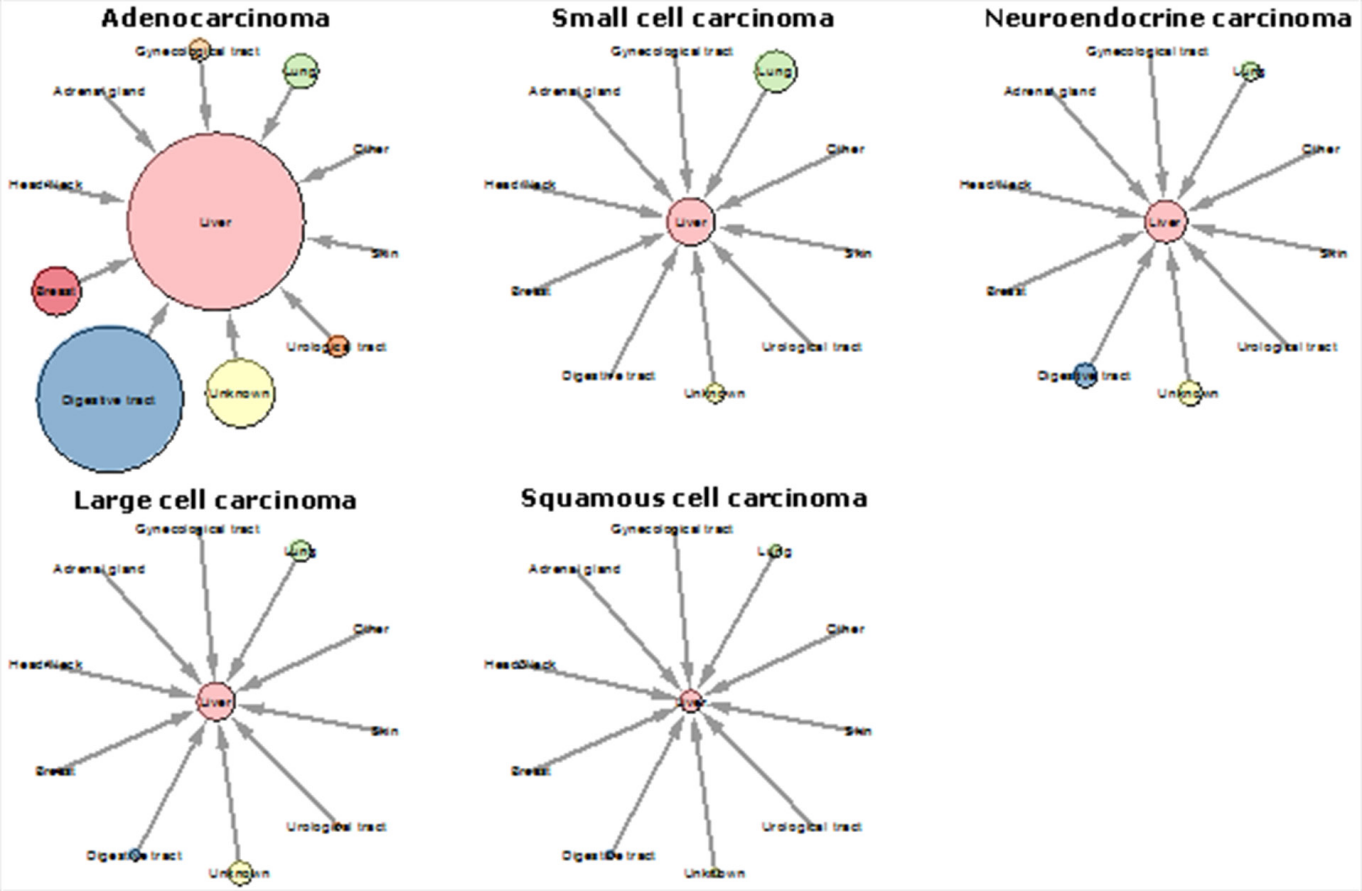
Origins of liver metastases of the carcinoma type Each clock plot shows the origins of liver metastases per carcinoma subtype (adenocarcinoma, small cell carcinoma, neuroendocrine carcinoma, large cell carcinoma, squamous cell carcinoma). Outer circles represent the location of the primary tumor. Circle size is proportional to the number of metastases.

The majority of adenocarcinoma N.O.S. originated from the digestive tract (*n*=11,829; 68.2%), especially from colorectal origin (*n*=8,004; 46.1%). In 2,709 patients (15.6%), the primary tumor location was not specified (Figure 1). For detailed information see Supplementary Table S2.

Liver metastases of the small cell subtype were most often of pulmonary origin (*n*=1043; 76.9%). Primary tumor location was not specified in 268 patients (19.7%). (Supplementary Table S2).

Neuroendocrine liver metastases originated most frequently from the digestive tract (*n*=389; 36.3%), especially from the pancreas (*n*=137; 12.8%) and duodenum (*n*=110; 10.3%). Pulmonary origin was observed in 238 patients (22.2%), and in 416 patients (38.8%) the primary tumor location was not specified (Supplementary Table S2, Figure 1).

The primary tumor in large cell carcinoma liver metastases was most frequently located in the lung (*n*=305; 34.8%), followed by digestive tract (*n*=97; 11.1%) and urological tract (*n*=41; 4.7%). Primary tumor location was not specified in 376 patients (42.9%) (Figure 1) (Supplementary Table S2).

Squamous cell carcinoma liver metastases originated most often from the lung (*n*=118; 35.2%) or the digestive tract (*n*=66; 18.6%), more specifically from the esophagus (*n*=39; 11.6%). In 44 patients (12.4%) the primary tumor was located in the oropharynx. Primary tumor location was not specified in 72 patients (21.5%) (Figure 1) (Supplementary Table S2).

### Melanoma

Metastatic melanoma was observed in 547 patients (2.4%) with liver metastases. Uveal melanoma was the primary tumor in 213 patients (38.9%), and primary cutaneous melanoma was the origin of liver metastases in 251 patients (45.8%). Liver metastases from mucosal melanoma were rare, with primary locations in the colon (*n*=3), small bowel (*n*=1), and urinary bladder (*n*=1). In 78 patients, the primary melanoma location was unknown (Supplementary Table S1).

### Sarcoma

Metastatic sarcoma was observed in 235 patients (1.0%) with liver metastases. The most prevalent subtype of metastatic sarcoma was gastrointestinal stromal tumor (GIST) (*n*=107; 45.5%), followed by leiomyosarcoma (*n*=64; 27.2%), and sarcoma N.O.S. (*n*=47; 20.0%) (Supplementary Table S1).

For GIST metastases, the following primary locations were described; colon or rectum (*n*=8), stomach (*n*=48), small bowel (*n*=22), and digestive tract N.O.S. (*n*=10). In 18 reports the primary GIST location was not specified.

Primary tumor locations in patients with metastatic leiomyosarcoma were: colon (*n*=1), stomach (*n*=4), small bowel (*n*=6), uterus (*n*=14), ovary (*n*=1), kidney (*n*=2), bone/soft tissue (*n*=33), or digestive tract (*n*=1). In 2 reports the location of the primary tumor location was not specified.

Primary tumor locations in patients with metastatic sarcoma N.O.S. were: bone/soft tissue (*n*=16); brain/meningeal (*n*=4); skin (*n*=2); rectum (*n*=1); oropharynx/nasopharynx (*n*=1) and small intestine (*n*=1). There was an unknown primary tumor location in 19 patients.

### Gender differences in primary tumor locations

**Figure 2:**
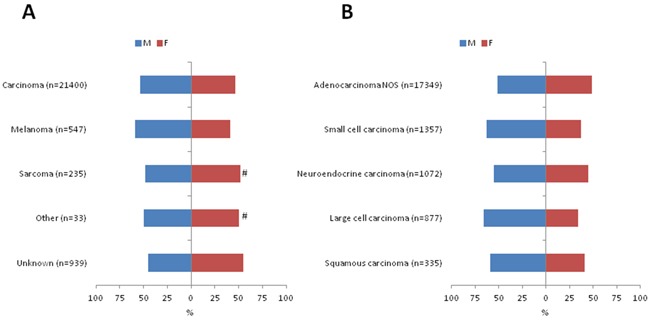
Differences in gender between tumor types A and the most important carcinoma subtypes **B.** #: no significant difference between women and men.

Histological confirmed liver metastases were more often observed in men than in women (*n*=12,280; 53.0% versus *n*=10,874; 47.0%; *p*<0.0001). Liver metastases from carcinoma (*n*=11,397; 53.3%; *p*=0.017), and melanoma (*n*=322; 58.9%; *p*=0.006) were more frequently diagnosed in men, whereas more women were diagnosed with liver metastases from an unknown tumor type (*n*=514; 54.8%; *p*<0.0001). In patients with liver metastases from carcinoma subtypes, male predominance was particularly observed in liver metastases with the subtypes: large cell carcinoma, small cell carcinoma, transitional carcinoma, and squamous cell carcinoma (Figure 2) (Supplementary Table S1).

Men presented relatively more often with liver metastases from colorectal adenocarcinoma compared to women [40.7% (*n*=4,625) versus 31.6% (*n*=3,379) (OR 1.63; 95%CI: 1.53-7.73; *p*<0.0001)].

Liver metastases from squamous cell lung carcinoma was more frequently observed in men than in women [45.7% (*n*=91) versus 19.9% (*n*=27) (95%CI: 2.052-5.638; *p*<0.0001)].

### Age differences

The majority of patients with liver metastases was older than 50 years (90.2%; *n*=20,892).

Metastatic adenocarcinoma from the digestive tract (including colorectal carcinoma liver metastases) were more common in older women (>70 years) than in younger women (≤50 [62.6% (*n*=1,823), versus 45.5% (*n*=540) (OR: 2.00; 95%CI: 1.75-2.30; *p<*0.0001)].

In contrast, a relative increased frequency of breast adenocarcinoma liver metastases was observed in younger women (≤50 years), compared to older women (>70 years) [34.2% (*n*=406) versus 8.9% (*n*=251) (OR 5.35; 95%CI 4.49-6.38; *p*<0.0001)]. There was no difference between young and older women in metastatic gynecological adenocarcinomas; 3.3% (age <50 years) versus 4.2% (age 51-70) versus 3.2% (age >70) (Figure 3A).

**Figure 3A:**
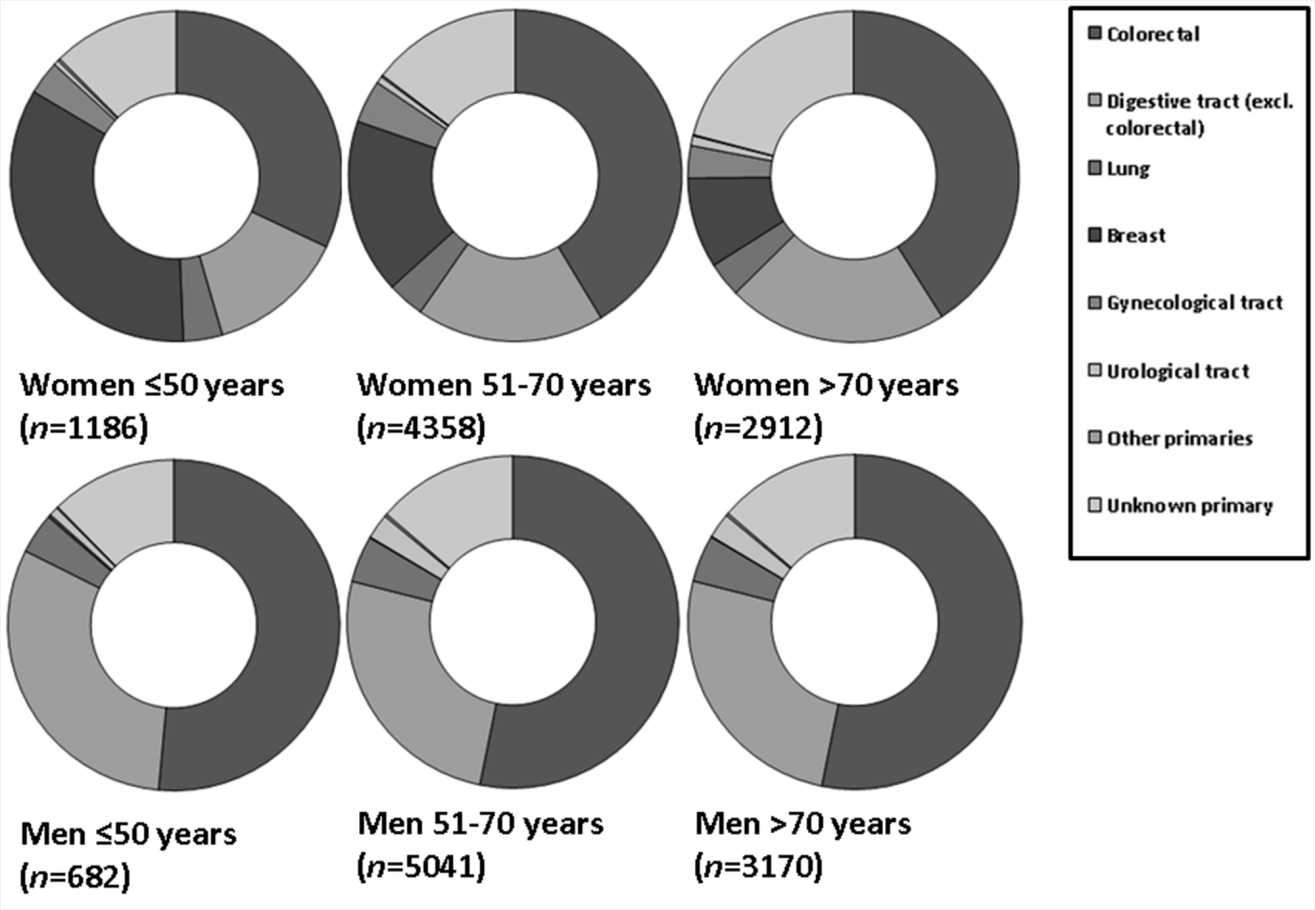
Relative incidences of primary tumor locations in women and men with metastatic adenocarcinoma In young women, metastatic breast cancer was more frequently observed (*p<*0.0001). Liver metastases from urological tumors were more frequently observed in men older than 50 years (*p*<0.0001).

However, metastatic squamous cell carcinomas and metastatic neuroendocrine carcinomas from the gynecologic tract were more frequently observed in young women (≤50 years, compared to women older than 50 years [38.9% (*n*=7) versus 16.1% (*n*=19) (OR: 4.13; 95%CI: 1.47-11.57; *p*=0.007) respectively, 4.8% (*n*=3) versus 1.0% (*n*=4) (OR: 5.19; 95%CI: 1.13-23.75; *p*=0.034)] (Figure 3B and 3C).

**Figure 3B:**
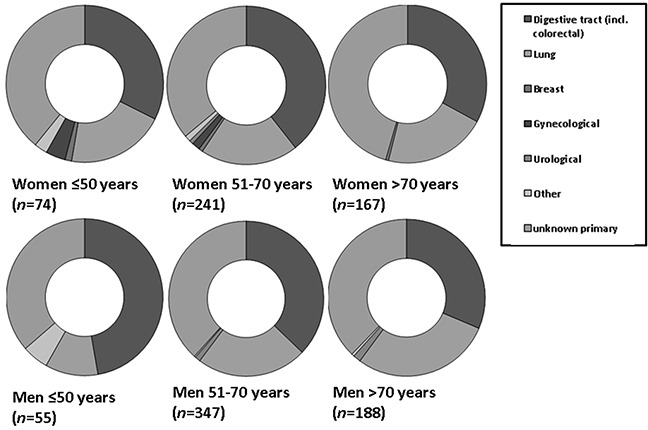
Relative incidences of primary tumor locations in women and men with metastatic neuroendocrine tumor In young women (≤50 years), the primary tumor was significantly more often located in the gynecological tract (*p*=0.034).

**Figure 3C:**
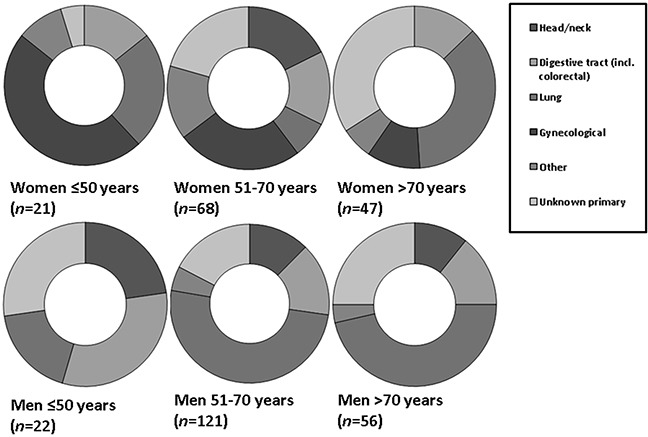
Relative incidences of primary tumor locations in women and men with metastatic squamous cell carcinoma Liver metastases from the gynecological tract were more frequently observed in women ≤ 50 years (*p*=0.007). Primary tumor location in the lung was observed significantly more frequent in men older than 50 years (*p*=0.41).

Metastatic adenocarcinoma originated relatively more frequent from urological tumors in men, and especially in men older than 50 years [3.1% (*n*=249) versus 0.9% in younger men (≤50 years) (*n*=6) (OR: 3.7; 95%CI: 1.51-8.94; *p*=0.04)] (Figure 3A).

In older men (>70 years), liver metastases from squamous cell lung carcinoma were more frequent than in younger men (≤50 years) [46.4% (*n*=26) versus 18.1% (*n*=4) (OR: 3.90; 95%CI: 1.17-13.00; *p*=0.027)]; liver metastases from squamous cell lung carcinoma were most common in middle aged male patients (51-70 years) (*n=*61; 50.4%) (Figure 3C).

### Unknown primary tumors

After reviewing all pathology history and follow up, there were 4,317 patients (18.6%) without a primary tumor location. In most of these unsolved cases, tumor type was reported (*n*=3,963; 91.8%). Carcinoma was the most common tumor type (*n*=3,847; 89.1%), and the most prevalent tumor subtype was adenocarcinoma N.O.S. (*n*=2,709; 62.8%).

There was a small male predominance in patients with an unknown primary (*n*=2,262; 52.4%).

Median age of patients with an unknown primary tumor was 68 years (range 0-96 years).

Patients with metastatic adenocarcinoma from an unknown primary tumor were significantly older at time of diagnosis, compared to patients with adenocarcinoma from a known primary tumor location; 68 years (range 25-96 years) versus 66 years (range 20-97 years) (*p*<0.0001). Median age of patients with an unknown primary from other tumor types and subtypes did not significantly differ from the age of patients with a known primary tumor.

## DISCUSSION

To the authors' knowledge, this is the first population-based study that describes the origins of histological confirmed liver metastases in a systematic way, including more than 23,000 patients during a 10-year time frame. The current study provides modern data on the origin and incidence of histological confirmed liver metastases from both biopsies and resection specimens in an era in which patients are treated according to modern standards.

Older cohort studies may represent a close approximation for the progression of untreated malignancies in humans [6], but it is of no doubt that in time metastatic patterns of various malignancies have changed. In the autopsy study by DiSibio et al. the primary tumors that spread most frequently to the liver included testicular cancer and breast cancer [6]. In that cohort testicular cancer spread to the liver in 75% of the patients, while in the present study the amount of liver metastases from testicular cancer was almost negligible, partly due to the fact that these tumors are currently diagnosed with markers in blood or by using imaging techniques [6]. Moreover, with the current treatment of resection and systemic therapy, the prognosis of testicular cancer improved tremendously and as a result, histological confirmed liver metastases are diagnosed less frequently nowadays [8, 9].

Reported incidences of breast cancer liver metastases also differed between autopsy studies and data of the present study [6, 10]. In time, a major improvement for breast cancer patients was made by the introduction of breast cancer screening programms [11], In addition, changes in chemotherapy, post-operative radiotherapy and hormonal therapy resulted in improved prognosis and led to decreased incidences in breast cancer liver metastases [6, 10, 12–14].

As to be expected, carcinoma was by far the most common tumor type (92%), more specifically adenocarcinoma N.O.S. (75%), found in patients with liver metastases. The most common primary tumor was colorectal carcinoma (35%). Liver resection was most often performed in patients with metastatic colorectal cancer, as was recently reported [15].

In general, gender distribution of the primary tumors corresponded with gender distribution of the liver metastases, although some remarkable differences were observed. Liver metastases from thyroid cancer were more frequently diagnosed in male patients (51.3%) while, according to the Dutch national cancer registry, primary thyroid carcinoma is more prevalent in women (approximately 73% of all thyroid cancer types). Despite the small number of patients with liver metastases from thyroid cancer, this might suggest that the behavior of thyroid carcinoma in male patients is more aggressive, which is confirmed by a worse prognosis in male patients [16]. Similar findings were observed in male patients with liver metastases from cutaneous melanoma. Although primary cutaneous melanoma is more frequently observed in female patients (58.3%, according to the Dutch National Cancer Registry), male patients are more frequently diagnosed with liver metastases (59%). Again this seems to be the result of aggressive tumor behavior in male patients [17, 18].

The clinical value of the present data might be questioned, but they can be used in the process of clinical decision making. In patients with multiple primary tumors and liver metastases, an approximation of the relative frequency of liver metastases can be derived from the current study. This could guide additional diagnostic (e.g.biopsy, immohistochemistry) or treatment strategies (e.g. surgery, systemic therapy).

The current overview can also be used for clinical decision making in patients with cancer of an unknown primary tumor (CUP). CUP is defined as a presentation of histologically confirmed metastases, where, despite a standardized diagnostic approach, no primary tumor can be detected [19]. In 24%-50% of the patients with CUP liver metastases are found [20–22]. Understanding the pathophysiological and molecular biology is needed to improve selective treatment strategies, based on the primary tumor and in the end to improve survival in patients with CUP. The current large dataset might be a basis for further research in this group of patients.

Despite the size of this large, nation-wide population based study, selection bias should not be underestimated. Obviously, not all patients with liver metastases will undergo liver biopsy. Especially in case of colorectal cancer liver metastases, the start of systemic treatment is often based on radiological diagnosis (CT-scan or FDG-PET scan) rather than on histological confirmation. Furthermore, although the liver is usually an easy access for biopsy, it is possible that histological or cytological confirmation was obtained from other metastatic sites such as: lymph nodes, ascites, pleural fluid, pulmonary lesions or any other metastatic location. Since these data and treatment, other than surgery, were not available in the current study, data on survival were not reported. On the other hand, many studies describe excellent results in patients diagnosed with liver metastases who underwent liver resection. This is not only the case in patients with colorectal cancer liver metastases, but improved survival has been reported in patients with other primary tumors, such as: breast cancer, melanoma, GIST or renal cell carcinoma [23–26].

In conclusion, this study provides an overview of the origins of liver metastases, with regard to tumor type, age and gender, in an era of modern diagnostic and treatment modalities. These important data form a basis for future research, and can be used for the development of diagnostic strategies.

## MATERIALS AND METHODS

### Patients and data collection

Data were collected using a search question with keywords in the PALGA-database; the nation-wide network and registry of histo-and cytopathology in the Netherlands. This network registers all pathology reports since 1971, with a nation-wide coverage since 1991 [27]. With the key words; “liver metastases”, “histology”, and limited to the years “2001-2010”, all pathology reports describing liver metastases were identified.

Pathology reports were excluded when patients underwent a liver resection or liver biopsy for a benign liver condition or for a primary malignant liver tumor such as hepatocellular carcinoma.

Per patient the following characteristics were collected from the pathology report: age and gender, the year of first histological diagnosis (in case of multiple biopsies, or a biopsy prior to liver resection), tumor type and subtype, and the location of the primary tumor.

For age three categories were used: 50 years and younger; between 51 and 70 years; and over 71 years. The patient's age at the first time of histological diagnosis was used in the analysis.

Tumor type and subtype were defined according to the International Classification of Disease (ICD-10). When the origin of the primary tumor was not described in the conclusion of the pathology report, additional reports of that patient were collected and evaluated to identify the primary tumor. When, after this assessment, no primary tumor could be detected, the primary tumor was classified as an “unknown primary”. Anonymous data were used and both the privacy committee and scientific committee of PALGA approved the study design.

### Statistical analysis

The chi-square test was used to compare nominal variables and the Mann-Whitney U test was used to compare continuous variables. A *p*-value of less than 0.05 was considered to be statistically significant. Multivariate regression analyses were used to determine differences in primary tumor locations between men and women in the age categories. All descriptive and statistical analyses were performed using statistical package for social sciences (SPSS, INC., Chicago, Illinois, USA version 18.0). Cytoscape version 3.2.1. was used to perform clock plots to visualize the origins of liver metastases of the carcinoma type. Circle size is the square root of the total number of liver metastases.

## SUPPLEMENTARY TABLES




