# Supplementary material for: Incidence and origin of histologically confirmed liver metastases: an explorative case-study of 23,154 patients
Source: Oncotarget. 2016 Jul 13;7(34):55368–76. doi: 10.18632/oncotarget.10552 (PMC5342423; doi:10.18632/oncotarget.10552)
Supplement: Supplementary file 1 [file oncotarget-07-55368-s001.docx]

| **Supplemental table 1**: Tumor types and sub-types of liver metastases diagnosed by pathological evaluation. N.O.S.: not otherwise specified |
| --- |

| **Tumor type** | **Sub-type** | **N**  **(%)** | **Sex**  **M (%) F (%)** | | **Median age y**  **(range)** |
| --- | --- | --- | --- | --- | --- |
| **Carcinoma** | **Total:** | 21400  (92.4%) | 11397  (53.3%) | 10003  (46.7%) | 66  (17-97) |
|  | Large cell carcinoma | 877 (3.7%) | 578 (65.9%) | 299 (34.1%) | 68 (1-90) |
|  | Small cell carcinoma | 1357 (5.9%) | 851 (62.7%) | 506 (37.3%) | 69 (25-91) |
|  | Squamous carcinoma | 335 (1.4%) | 199 (59.4%) | 136 (40.6%) | 65 (27-88) |
|  | Transitional carcinoma | 262 (1.2%) | 199 (76.0%) | 63 (24.0%) | 69 (41-89) |
|  | Adenocarcinoma N.O.S. | 17349 (74.9%) | 8892 (51.3%) | 8457 (48.7%) | 66 (20-97) |
|  | Adenoid cystic carcinoma | 5 (0%) | 1(20%) | 4(80%) | 58 (40-63) |
|  | Neuroendocrine carcinoma | 1072 (4.6%) | 590 (55.0%) | 482 (45%) | 65 (17-96) |
|  | Merkel cell carcinoma | 8 (0%) | 6 (71.4%) | 2(28.6%) | 72 (48-82) |
|  | Renal cell carcinoma | 102 (0.4%) | 67 (65.7%) | 35 (34.3%) | 68 (37-87) |
|  | Medullary carcinoma | 16 (0.1%) | 11 (68.8%) | 5 (31.3%) | 49 (17-73) |
|  | Acinic cell carcinoma | 1 (0%) | 1(100%) | 0 | 31 |
|  | Thymic carcinoma | 4 (0%) | 2(50%) | 2 (50%) | 53 (35-59) |
|  | Granulosacell carcinoma | 11 (0.0%) | 0 | 11 (100%) | 53 (39-71) |
|  | Malignant mixed mullerian carcinoma | 1 (0%) | 0 | 1(100%) | 79 |
| **Melanoma** | **Total:** | 547  (2.4%) | 322  (58.9%) | 225  (41.1%) | 63  (20-88) |
|  | Uveal | 213 (0.9%) | 123 (57.7%) | 90 (42.3%) | 65 (30-88) |
|  | Cutaneous | 251 (1.1%) | 148 (59.0%) | 103 (41.0%) | 63 (20-87) |
|  | Mucosal | 5 (0%) | 3 (60.0%) | 2 (40.0%) | 66 (51-80) |
|  | Unknown primary | 78 (0.3%) | 48 (61.5%) | 30 (38.5%) | 61 (26-87) |
| **Sarcoma** | **Total:** | 235  (1.0%) | 113  (48.5%) | 122  (51.5%) | 60  (3-86) |
|  | Sarcoma N.O.S. | 47 (0.2%) | 21 (44.7%) | 26 (55.3%) | 61 (30-85) |
|  | GIST | 107 (0.5%) | 63 (58.9%) | 44 (41.1%) | 62 (22-86) |
|  | Angiosarcoma | 3 (0%) | 1 (33.3%) | 2 (67.7%) | 57 (3-75) |
|  | Liposarcoma | 3 (0%) | 3 (100%) | 0 | 59 (37-72) |
|  | Leiomyosarcoma | 64 (0.2%) | 22 (34.4%) | 42 (65.6%) | 59 (33-85) |
|  | Rhabdomyosarcoma | 1 (0%) | 0 | 1 (100%) | 13 |
|  | Synoviasarcoma | 2 (0%) | 1 (50%) | 1 (50%) | 52 (41-93) |
|  | Osteosarcoma | 1 (0%) | 1 (100%) | 0 | 61 |
|  | Chondrosarcoma | 2 (0%) | 0 | 2 (100%) | 72 (58-86) |
|  | Ewing sarcoma | 1 (0%) | 0 | 1 (100%) | 14 |
|  | Hemangiopericytoma | 4 (0%) | 1 (25%) | 3 (75%) | 45 (31-66) |
| **Other** | **Total** | 33  (0.1%) | 22  (67.7%) | 11  (33.3%) | 45  (0-76) |
|  | Neuroblastoma | 6 (0%) | 1(16.6%) | 5(83.3%) | 0 (0-1) |
|  | Nefroblastoma | 3 (0%) | 2 (67.7%) | 1 (33.3%) | 6 (5-37) |
|  | Mesothelioma | 6 (0%) | 5(83.3%) | 1(16.7%) | 70 (55-76) |
|  | Chordoma | 1 (0%) | 1(100%) | 0 | 72 |
|  | Ameloblastoma | 1 (0%) | 0 | 1(100%) | 50 |
|  | Insulinoma/glucagonoma | 2 (0%) | 1(50%) | 1(50%) | 65 (55-74) |
|  | Brenner tumor | 1 (0%) | 0 | 1(100% | 69 |
|  | Non-seminoma | 2 (0%) | 2 (100%) | 0 | 26 (17-35) |
|  | Germcell carcinoma | 9 (0%) | 8 (88.9%) | 1 (11.1%) | 33(18-78) |
|  | Choriocarcinoma | 2 (0%) | 2(100%) | 0 | 36 (22-50) |
| **Unknown tumor type** |  | 939  (4.1%) | 425  (45.2%) | 514  (54.8%) | 66  (1-90) |
| **Total:** |  | 23154  (100%) | 12279  (53.0%) | 10875  (47%) | 66  (0-97) |
|  | | | | | |
