# Supplementary material for: Incidence and origin of histologically confirmed liver metastases: an explorative case-study of 23,154 patients
Source: Oncotarget. 2016 Jul 13;7(34):55368–76. doi: 10.18632/oncotarget.10552 (PMC5342423; doi:10.18632/oncotarget.10552)
Supplement: Supplementary file 2 [file oncotarget-07-55368-s002.docx]

**Supplemental table 2:** Primary tumor locations in patients with histological confirmed liver metastases from adenocarcinoma; small cell carcinoma; neuroendocrine carcinoma; large cell carcinoma and squamous cell carcinoma. N.O.S.: not otherwise specified.

|  |  | **Adeno-**  **carcinoma** | **Small cell carcinoma** | **Neuroendocrine carcinoma** | **Large cell carcinoma** | **Squamous cell carcinoma** |
| --- | --- | --- | --- | --- | --- | --- |
| **Head/Neck** | Total: | 19  (0.1%) | - | 2  (0.2%) | 23  (2.6%) | 46  (13.0) |
|  | Pharynx/Larynx | 8 (0.0%) | - | - | 18 (2.1%) | 44 (12.4%) |
|  | Thymus | 1 (0.0%) | - | - | 3 (0.3%) | 1 (0.3%) |
|  | Thyroid gland | 10 (0.1%) | - | 2 (0.2%) | 2 (0.2%) | 1 (0.3%) |
| **Digestive Tract** | Total: | 11829  (68.2%) | 24  (1.8%) | 389  (36.3%) | 97  (11.1%) | 66  (18.6%) |
|  | Colon/Rectum/Appendix | 8004 (46.1%) | 11 (0.8%) | 86 (8.0%) | 21 (2.4%) | 7 (2.1%) |
|  | Anus | 2 (0.0%) | - | 1 (0.1%) | 1 (0.1%) | 14 (4.2%) |
|  | Stomach | 507 (2.9%) | 3 (0.2%) | 13 (1.2%) | 9 (1.0%) | 1 (0.3%) |
|  | Esophagus | 349 (2.0%) | 2 (0.1%) | 3 (0.3%) | 15 (1.7%) | 39 (11.6%) |
|  | Gall bladder/biliary tract | 237 (1.4%) | 2 (0.1%) | 1 (0.1%) | 7 (0.8%) | 2 (0.6%) |
|  | Pancreas | 1755 (10.1%) | 5 (0.4%) | 137 (12.8%) | 28 (3.2%) | 2 (0.6%) |
|  | Duodenum/small intestine | 76 (0.4%) | - | 110 (10.3%) | 1 (0.1%) | 1 (0.3%) |
|  | N.O.S. | 899 (5.2%) | 1 (0.1%) | 38 (3.5%) | 15 (1.7%) | - |
| **Lung** |  | 731  (4.2%) | 1043  (76.9%) | 238  (22.2%) | 305  (34.8%) | 118  (35.2%) |
| **Skin** |  | - | - | 1 (0.1%) | 1 (0.1%) | 6 (1.8%) |
| **Breast** |  | 1417 (8.2%) | 2 (0.1%) | 4 (0.4%) | 24 (2.7%) | - |
| **Gynecological tract** | Total: | 314 (1.8%) | 5  (0.3%) | 7  (0.7%) | 8  (0.9%) | 26  (7.8%) |
|  | Ovary | 212 (1.2%) | 3 (0.2%) | 5 (0.5%) | 3 (0.3%) | - |
|  | Uterus | 87 (0.5%) | 2 (0.1%) | 1 (0.1%) | 1 (0.1%) | 2 (0.6%) |
|  | Cervix | 15 (0.1%) | - | 1 (0.1%) | 4 (0.5%) | 24 (7.2%) |
| **Urological tract** | Total: | 319  (1.8%) | 15  (1.1%) | 10  (1.0%) | 41  (4.7%) | 1  (0.3%) |
|  | Kidney | 144 (0.8%) | - | 2 (0.2%) | 9 (1.0%) | - |
|  | Urinary bladder | 20 (0.1%) | 8 (0.6%) | 3 (0.3%) | 23 (2.7%) | 1 (0.3%) |
|  | Prostate | 154 (0.9%) | 7 (0.5%) | 5 (0.5%) | 7 (0.8%) | - |
|  | Testis | 1 (0.0%) | - | - | 2 (0.2%) | - |
| **Adrenal** |  | 6 (0.0%) | - | 5 (0.5%) | 2 (0.2%) | - |
| **Other** |  | 5 (0.0%) | - | - | - | - |
| **Unknown primary** |  | 2709 (15.6%) | 268 (19.7%) | 416 (38.8%) | 376 (42.9%) | 72 (21.5%) |
| **Total** |  | **17349** | **1357** | **1072** | **877** | **335** |
|  | | | | | | |
